# Serological Studies Confirm the Novel Astrovirus HMOAstV-C as a Highly Prevalent Human Infectious Agent

**DOI:** 10.1371/journal.pone.0022576

**Published:** 2011-08-04

**Authors:** Peter D. Burbelo, Kathryn H. Ching, Frank Esper, Michael J. Iadarola, Eric Delwart, W. Ian Lipkin, Amit Kapoor

**Affiliations:** 1 Neurobiology and Pain Therapeutics Section, Laboratory of Sensory Biology, National Institute of Dental and Craniofacial Research, National Institutes of Health, Bethesda, Maryland, United States of America; 2 Department of Pediatrics, University Hospitals Case Medical Center, Cleveland, Ohio, United States of America; 3 Blood Systems Research Institute and Department of Laboratory Medicine, University of California San Francisco, San Francisco, California, United States of America; 4 Center for Infection and Immunity, Columbia University, New York, New York, United States of America; College of Medicine, Hallym University, Korea

## Abstract

Molecular identification of a microbe is the first step in determining its prevalence of infection and pathogenic potential. Detection of specific adaptive immune responses can provide insights into whether a microbe is a human infectious agent and its epidemiology. Here we characterized human anti-IgG antibody responses by luciferase immunoprecipitation systems (LIPS) against two protein fragments derived from the capsid protein of the novel HMOAstV-C astrovirus. While antibodies to the N-terminal fragment were not informative, the C-terminal capsid fragment of HMOAstV-C showed a high frequency of immunoreactivity with serum from healthy blood donors. In contrast, a similar C-terminal capsid fragment from the related HMOAstV-A astrovirus failed to show immunoreactivity. Detailed analysis of adult serum from the United Sates using a standardized threshold demonstrated HMOAstV-C seropositivity in approximately 65% of the samples. Evaluation of serum samples from different pediatric age groups revealed that the prevalence of antibodies in 6–12 month, 1–2 year, 2–5 year and 5–10 year olds was 20%, 23%, 32% and 36%, respectively, indicating rising seroprevalence with age. Additionally, 50% (11/22) of the 0–6 month old children showed anti-HMOAstV-C antibody responses, likely reflecting maternal antibodies. Together these results document human humoral responses to HMOAstV-C and validate LIPS as a facile and effective approach for identifying humoral responses to novel infectious agents.

## Introduction

The family *Astroviridae* consists of small (28–30 nm in diameter), non-lipid enveloped, single-stranded positive-sense RNA viruses with genomes ranging in size from 6.4 to 7.3 kb. The genome includes three open reading frames (ORFs) designated ORF1a, ORF1b and ORF2. ORF1a encodes the non-structural polyprotein 1a, while the longer ORF1b encodes polyprotein 1ab including the RNA dependent RNA polymerase (RdRp) expressed through a ribosomal frameshift at the ORF1a/1b junction. ORF2 encodes the viral capsid structural polyprotein [1], [2]. To date the *Astroviridae* family consists of two genera, *Astrovirus* and *Mamastrovirus*, which infect avian and mammalian hosts, respectively. These astroviruses, transmitted through the fecal-oral route can cause gastroenteritis in numerous avian and mammalian species, including humans [3], [4]. All eight known human astrovirus serotypes belonging to the first identified human Astrovirus species (HAstV) have been associated with gastroenteritis [5], [6], [7], [8], [9]. Clinical symptoms of HAstV infection in humans usually lasts between two and four days [10] and consists of watery diarrhea and less commonly, vomiting, headache, fever, abdominal pains and anorexia. HAstV can also cause significant disease in the elderly and immunocompromised patients [11].

Several metagenomics studies have recently used random amplification and mass sequencing of nucleic acid extracted from human stool to systematically catalogue viruses, phage and bacteria present in patients with diarrhea [12], [13], [14], [15]. For example, a novel astrovirus species, AstV-MLB1, was identified in stool from children and adults, including some with diarrhea [13], [16], [17], [18]. In addition, a new group of astroviruses, provisionally named HMOAstV/AstV-VA, were discovered by consensus PCR using stool samples from different continents [13], [18]. Phylogenic analysis of the HMOAstV viruses revealed that they consisted of three subgroups, HMOAstV-A, HMOAstV-B, and HMOAstV-C. HMOAstV-C RNA was also identified in individuals from a gastroenteritis outbreak in a daycare center in Virginia and tentatively named AstV-VA1 [19]. Despite the identification of novel astroviruses in human stool, it is unclear if these new viruses are pathogenic or whether they are passengers associated with ingested food.

Our objective was to determine the seroprevalence of anti-HMOAstV-C antibodies in children and adults in the US. Traditionally, ELISA assays are used for serological testing and require purified virus or recombinant viral proteins. Unfortunately, ELISA tests often show cross-reactivity, have inherent high background signals and require extensive optimization and standardization. Luciferase Immunoprecipitation System (LIPS) is a new technology that employs luciferase-tagged antigens in a liquid phase immunoprecipitation assay [20]. LIPS offers several advantages over ELISA including low backgrounds, highly quantitative data and the ability to generate diagnostically useful serodeterminations without pre-determined cut-off values [20], [21]. To determine if the novel HMOAstV-C astrovirus is a prevalent human virus, LIPS was used to screen for antibodies against conserved regions of the HMOAstV-C viral capsid.

## Materials and Methods

### Serum samples

Serum samples from adult, healthy blood donors (n = 106) were collected without any clinical information under IRB approved protocols at the National Institutes of Health, Bethesda, Maryland. Children serum samples were from Rainbow Babies and Children's Hospital, Cleveland, Ohio and obtained under IRB approval from University Hospitals – Case Medical Center. A total of 103 serum samples from children from different ages were analyzed as follows: 0-6 months (n = 22), 6–12 months (n = 15), 1-2 years (n = 22), 2-5 years (n = 22) and 5-10 years old (n = 22). Samples were collected from September 2009 through March 2010. Other than the age of the individual from whom the serum was obtained, no other clinical information was available.

Based on the similarity of HMOAstV-C astrovirus with animal astroviruses [13], serum samples from different domesticated animals, including horses, pigs and rabbits, were tested. Pig and horse serum samples were the kind gift of Yanjin Zhang and Utpal Pal (VA-MD Regional College of Veterinary Medicine, Univ. of Maryland). Rabbit serum samples were obtained from NIH laboratories and commercial vendors. All serum samples were stored at −80°C, thawed, and then left at 4°C prior to processing for LIPS analysis.

### Generation of Ruc-Astrovirus antigen fusion constructs

Templates for capsid coding sequences of HMOAstV-C and HMOAstV-A were generated by RT-PCR amplification using human stool as described [13]. Due to the possibility of antibody cross-reactivity to different regions of the HMOAstV-C capsid, two different fragments encompassing the N-terminal (amino acids 1-393) and C-terminal fragment (amino acids 402-704) were generated by PCR. The primer adapter sequences used to clone each protein fragment are as follows: N-terminal capsid fragment of HMOAstV-C, 5′-GAGGGATC CATGGCTGGTAAACAGCCC-3′ and 5′-GAGCTCGAGTCAAGGGCCTGTGTTAGGTGC-3′ and C-terminal capsid fragment of HMOAstV-C, 5′- GAGGGATCCAACACCACTACAGGGTCA-3′ and 5′-GAGCTCGAGTCAATCCAGTGGGGTCAATCT-3′. A third C-terminal capsid fragment from the HMOAstV-A astrovirus (amino acids 396-700), spanning a similar region to HMOAstV-C was constructed with the primers: 5′-GAGGGATCCAGCACAGCCTCAGCGG TT -3′ and 5′-GAGCTCGAGTCAATCCTTAGGCTTCTTTCT-3′. The three capsid fragments were subcloned downstream of *Renilla* luciferase (Ruc) using the pREN2 vector [22]. DNA sequencing was used to confirm the integrity of the three DNA constructs. The sequence for the C-terminal capsid fragment of HMOAstV-C has been deposited in GenBank with accession (JF313458). Plasmid DNA was then prepared from these two different pREN2 expression vectors using a Qiagen Midi preparation kit. Following transfection of mammalian expression vectors, crude protein extracts were obtained as described for use as antigen [23].

### LIPS assays

Briefly, human and animal sera were processed in a 96-well format at room temperature as previously described [23]. Serum samples were first diluted 1∶10 in assay buffer A (50 mM Tris, pH 7.5, 100 mM NaCl, 5 mM MgCl_2_, 1% Triton X-100) using a 96-well polypropylene microtiter plate. Antibody titers were measured by adding 40 µl of buffer A, 10 µl of diluted sera (1 µl equivalent), and 1×10^7^ light units (LU) of each of the Ruc-HMOAstV antigen fragments containing crude Cos1 cell extract to wells of a polypropylene plate and incubated for 60 minutes at room temperature on a rotary shaker. Next, 5 µl of a 30% suspension of Ultralink protein A/G beads (Pierce Biotechnology, Rockford, IL) in PBS were added to the bottom of each well of a 96-well filter HTS plate (Millipore, Bedford, MA). To this filter plate, the 100 µl antigen-antibody reaction mixture was transferred and incubated for 60 minutes at room temperature on a rotary shaker. The washing steps of the retained protein A/G beads were performed on a Biomek Workstation or Tecan plate washer with a vacuum manifold. After the final wash, LU were measured in a Berthold LB 960 Centro microplate luminometer (Berthold Technologies, Bad Wilbad, Germany) using coelenterazine substrate mix (Promega, Madison, WI). All LU data were obtained from the average of at least two separate experiments.

### Sequence analyses

Using the C-terminal capsid fragment of HMOAstV-C as the query sequence, a BLAST search was performed against the non-redundant NCBI protein databases. From this analysis, the highest homology was with HMOAstV-B and HMOAstV-A astroviruses. Viral capsid sequences were aligned using the global alignment program COBALT (www.ncbi.nlm.nih.gov/guide/sequence-analysis/) with default parameters.

### Data analysis

GraphPad Prism software (San Diego, CA) was used for statistical analysis. For the calculation of sensitivity and specificity, a cut-off limit was used, which was derived from the combined value of the mean value plus 3 standard deviations (SD) of the replica samples containing only buffer, Ruc-extract and protein A/G beads. Human blood donor samples highly positive for anti-HMOAstV-C antibodies were used as internal positive controls to standardize the LIPS parameters for testing of serum samples.

## Results

### Identification of human antibody responses to the capsid of HMOAstV-C

**Figure 1:**
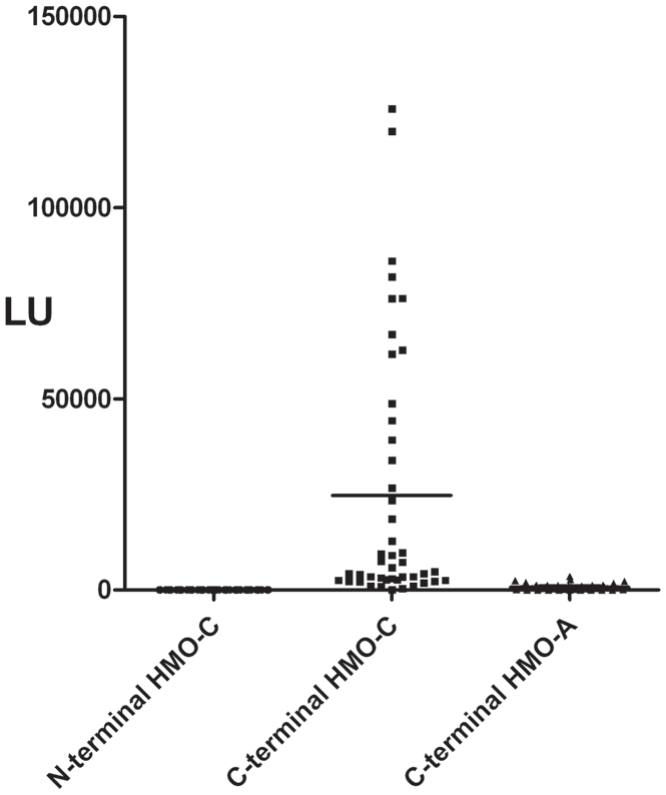
LIPS detection of antibodies to a C-terminal capsid fragment of HMOAstV-C. Antibodies to the N- and C-terminal capsid protein of HMOAstV-C and the C-terminal capsid protein of HMOAstV-CA capsid protein fragments were analyzed in 45 adult serum samples. Each symbol represents individual serum samples tested with each protein fragments and LU values were adjusted by subtracting background binding to protein A/G beads. The short solid line represents the mean antibody titer for each group.

While most *bona fide* antigenic targets used in LIPS assays show high sensitivity and specificity [20], the exact antigens useful for diagnosis of HMOAstV-C are not known. As a screening approach and to potentially eliminate cross-reactivity spanning the full-length capsid regions of these viruses, we chose to first test two different protein fragments encompassing conserved N- and C-terminal capsid fragments of HMOAstV-C. From LIPS screening of 45 adult blood donor samples, the HMOAstV-C N-terminal capsid fragment showed higher background binding to the mock protein A/G beads than to clinical samples and was judged not to be immunoreactive (Fig. 1). However, the C-terminal capsid fragment of HMOAstV-C protein demonstrated a robust response up to 125,900 LU above background (Fig. 1). The HMOAstV-C immunoreactivity in these human seropositive samples was highly reproducible and could be completely eliminated by pre-adsorption with protein A/G beads (data not shown). A third protein fragment derived from a similar C-terminal capsid region of the related HMOAstV-A virus failed to show immunoreactivity with any of the human samples tested (Fig.1). These results suggest that detectable human antibody responses are specific for HMOAstV-C and not for HMOAstV-A astrovirus.

**Figure 2:**
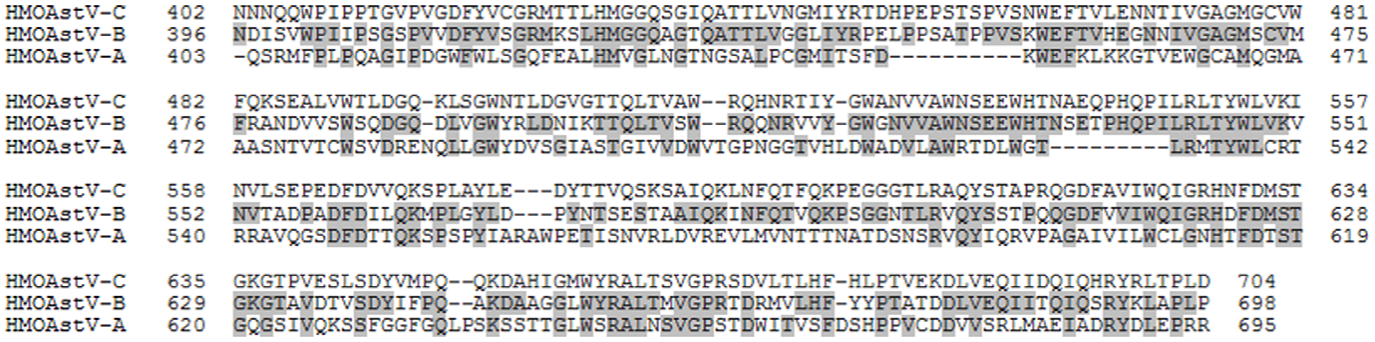
Comparison of the C-terminal capsid fragment of HMOAstV-C with related viruses. From BLASTP analysis, the highest homology of the HMOAstV-C capsid fragment was with HMOAstV-B (68% identity) and HMOAstV-A (30% identity) astroviruses. Identical amino acid residues with HMOAstV-C are shaded.

Sequence analysis of the C-terminal fragment of HMOAstV-C revealed 68% and 30% amino acid identity with corresponding capsid region from the novel Astrovirus, HMOAstV-B and HMOAstV-A astroviruses, respectively (Fig. 2). Importantly, no significant homology of the HMOAstV-C protein fragment was detected with the capsids derived from the eight known serotypes of HAstV strains (data not shown).

### High prevalence of anti-HMOAstV-C antibodies in adult humans

**Figure 3:**
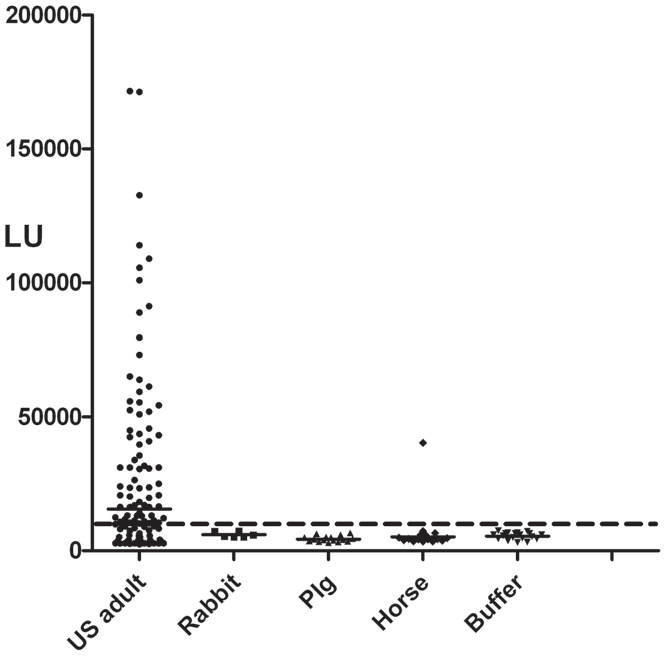
LIPS detection of antibodies to the C-terminal capsid fragment of HMOAstV-C in human and animal serum samples. Immunoreactivity to the HMOAstV-C was determined in 106 healthy adult US blood donors, 6 rabbits, 16 horses, 16 pigs and 14 buffer only controls. Raw LU values are shown without subtracting background binding to protein A/G beads. The short solid line represents the mean titer for each group. The dashed line represents the diagnostic cut-off, derived from the mean plus 3 SD of 19 replica buffer blank samples.

To determine if HMOAstV-C infection is unique to humans, additional LIPS analysis was performed to compare immunoreactivity in animal and human serum samples. For these studies, immunoreactivity against the C-terminal capsid fragment of HMOAstV-C was examined in 106 healthy adult US blood donors side-by-side serum samples from different domesticated animals. Buffer blanks in the LIPS format were used as negative controls to evaluate seropositivity [24] and a cut-off value was calculated from the mean plus 3 SD of 19 replica samples containing only buffer, Ruc-extract and protein A/G beads. Using a threshold of >9,525 LU, 65% (69 of 106) of healthy adult US blood donors were seropositive (Fig. 3). In contrast, no immunoreactivity was detected in any of the rabbit (n = 6), or pig (n = 16) serum samples (Fig. 3). Of 16 horse serum samples, one sample (6.25%) was immunoreactive to HMOAstV-C antigen (Fig. 3). As a control, several antigens were tested and revealed highly robust antibody titers in these same animal serum samples against known animal pathogens (data not shown). Collectively, these results suggest that antibodies against HMOAstV-C are relatively common in human adults, but are rare or non-existent in pigs, rabbits and horses.

### Increased exposure to HMOAstV-C with age

**Figure 4:**
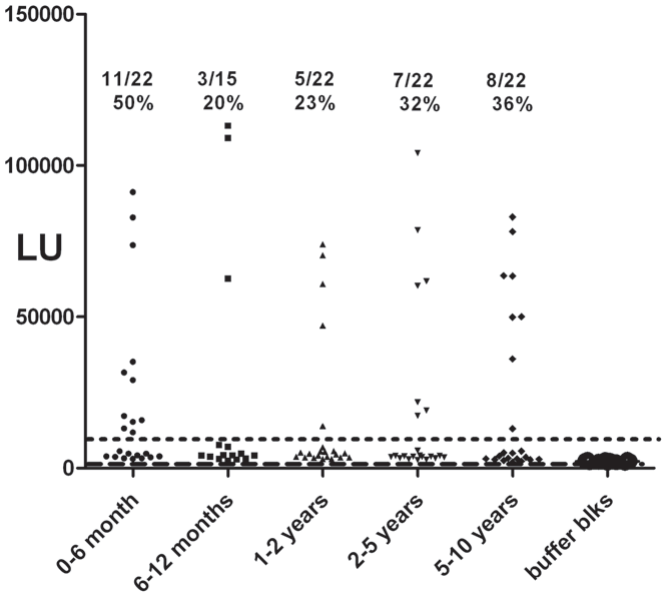
Prevalence of HMOAstV-C antibodies with childhood age. A total of 103 child serum samples were analyzed by LIPS including from the following age brackets: 0–6 months (n = 22), 6–12 month (n = 15), 1–2 year (n = 22), 2–5 year (n = 22), and 5–10 year olds (n = 22). Raw LU values are shown without subtracting background binding to protein A/G beads. The dashed line represents the diagnostic cut-off value derived from the mean plus 3SD of replica buffer blank samples. The fraction and percent seropositive for HMOAstV-C are shown above each group.

We next explored differences in HMOAstV-C seroprevalence amongst different aged children in the United States. LIPS analysis revealed that the prevalence of infection in 6–12 month, 1–2 year, 2–5 year and 5–10 year olds was 20%, 23%, 32% and 36%, respectively. These results suggest a trend of increased antibody prevalence with increasing age, although no statistical difference was observed between age groups (Fig. 4). Additionally, 50% (11/22) of the 0-6 month old children showed anti-HMOAstV-C antibody responses, which likely reflects the presence of maternal antibodies. No statistical difference in antibody titers was observed between these different groups of children. All of the different children age groups showed a significantly lower prevalence of HMOAstV-C antibodies than adults (Fischer Exact T test; *p*<0.005). These results are consistent with increased infection by HMOAstV-C over time.

## Discussion

Although several novel viruses have been identified in human stool [12], [13], [14], [15], few studies have examined humoral responses to them. We have established a robust, sensitive serology platform that is ideally suited for pathogen discovery applications. LIPS holds several advantages over ELISA for studying humoral responses against potentially new infectious agents stemming from the low background binding, high sensitivity innate to this assay and the ability to rapidly test different antigens/antigen fragments using a standard format with little or no assay optimization [20]. Here we used LIPS assays to demonstrate humoral responses to the HMOAstV-C Astrovirus capsid protein and provide experimental data supporting human infection.

Several different lines of evidence indicate that the HMOAstV-C capsid fragment is a target of humoral responses. First, only the C-terminal capsid region of HMOAstV-C was immunoreactive with human serum samples. Although RNA from HMOAstV-A was previously identified in human stool samples, we were unable to detect human immunoreactivity to this analogous C-terminal capsid region of HMOAstV-A. The exact reason for the lack of immunoreactivity is not clear, but it is possible that this capsid region of HMOAstV-A is less antigenic or its conformational folding is not recapitulated in the current *Renilla* luciferase fusion protein using the LIPS system. Second, the HMOAstV-C astrovirus capsid region used in LIPS was relatively unique and had no significant homology with the eight known serotypes of HAstV strains. Based on its homology, it is possible that some of the observed HMOAstV-C capsid immunoreactivity is against the related HMOAstV-B capsid. However, our previous LIPS studies demonstrate markedly different serologic responses to similarly homologous antigens in subjects infected with related pathogens [25], [26]. In these studies, variations in both linear and conformational epitopes are more clearly differentiated using the LIPS liquid assay than using ELISAs [20]. Further studies examining the humoral responses against the HMOAstV-B capsid, as well as additional proteins from the HMOAstV viruses should resolve this issue. Third, the anti-HMOAstV-C capsid antibody titers detected by LIPS assay in human samples was substantially higher than the background binding associated with mock protein A/G beads alone. This magnitude is comparable to those seen by LIPS against other infection agents such as *Borrelia burgdorferi* [27] and Kaposi Sarcoma associated virus [28]. Our strategy of using buffer blanks instead of seronegative uninfected samples has been used in other seroepidemiologic investigations and provide comparable diagnostic thresholds as seronegative samples [24], [29]. Fourth, the relatively low immunoreactivity against HMOAstV-C in young children (age 6-12 months) and a corresponding increase in seroprevalence with childhood age is consistent with human infection. Lastly, additional studies evaluating rabbit, pig and horse samples showed that anti-HMOAstV-C antibodies were rare or absent in animals. The lack of immunoreactivity against HMOAstV-C in animals, despite the greater homology of HMOAstV-C to animal Astroviruses [13], supports the notion that humans are a host for these viruses. The approach of analyzing humoral responses to a panel of different animal serums side-by-side human samples by LIPS is useful for understanding the host range for this and other potential pathogens.

Overall the results suggest that HMOAstV-C is a common infectious agent circulating in human populations. It is also possible that the studies described here underestimate the true seroprevalence of HMOAstV-C that may result from transient antibody titers from older infections and the genetic diversity of this newly recognized agent. The higher prevalence of antibodies in adults (65%) suggests that most adults are seropositive; however, it is unknown whether the presence of antibody is protective against infection. Nevertheless, compared to children, adults show a markedly higher incidence of HMOAstV-C infection. Furthermore, the detection of high prevalence of antibodies in the 0-6 month old children is consistent with the presence of maternal antibodies. In infants, waning maternal antibodies for many infectious agents is often observed [30], [31].

Additional investigation including prospective analysis of antibody responses by LIPS from acute infection would be worthwhile. It is interesting to note that HMOAstV-C/AstV-VA1 was identified as an Astrovirus associated with an outbreak of gastroenteritis in a child daycare center in Virginia [19]. RT-PCR and sequencing of affected individuals showed identical HMOAstV-C sequences. It is highly likely that there are diverse HMOAstV-C strains causing a spectrum of clinical symptoms. Along these lines, an Astrovirus, phylogenetically related to HMOAstV-C (95% identical capsids) was recently detected in the brain of a child with encephalitis who had a genetic form of agammaglobulinemia [32]. Further studies are clearly needed to establish the clinical spectrum of disease caused by HMOAstV-C. Although a single antigenic capsid target was diagnostically useful here with HMOAstV-C, the ability of detecting antibody responses to multiple independent protein fragments can increase assay sensitivity and has the potential to stratify different conditions caused by the same infectious agent [20]. The development of *Renilla* luciferase antigen fusions for additional HMOAstV-C Astrovirus proteins and other Astrovirus in the LIPS system will likely be useful for identifying and understanding their role in diarrheal and other illnesses.
